# Correlation between Lamina Cribrosa Tilt Angles, Myopia and Glaucoma Using OCT with a Wide Bandwidth Femtosecond Mode-Locked Laser

**DOI:** 10.1371/journal.pone.0116305

**Published:** 2014-12-31

**Authors:** Takuhei Shoji, Hiroto Kuroda, Masayuki Suzuki, Motoyoshi Baba, Masanori Hangai, Makoto Araie, Shin Yoneya

**Affiliations:** 1 Department of Ophthalmology, Saitama Medical University, Iruma, Saitama, Japan; 2 Advanced Laser Medical Center, Department of Ophthalmology, Saitama Medical University, Iruma, Saitama, Japan; 3 Department of Ophthalmology, Kanto Central Hospital, Tokyo, Japan; Medical College of Soochow University, China

## Abstract

**Purpose:**

To measure horizontal and vertical lamina cribrosa (LC) tilt angles and investigate associated factors using prototype optical coherence tomography (OCT) with a broad wavelength laser light source.

**Design:**

Cross sectional study.

**Methods:**

Twenty-eight no glaucoma eyes (from 15 subjects) and 25 glaucoma eyes (from 14 patients) were enrolled. A total of 300 optic nerve head B-scans were obtained in 10 µm steps and the inner edge of Bruch's membrane opening (BMO) was identified as the reference plane. The vertical and horizontal angles between BMO line and approximate the best-fitting line for the surface of the LC were measured and potential associated factors were estimated with univariate and multivariate logistic regression analyses.

**Results:**

The median (interquartile range) horizontal and vertical tilt angles were 7.10 (2.43–11.45) degrees and 4.15 (2.60–6.85) degrees in eyes without glaucoma and 8.50 (4.40–14.10) degrees and 9.30 (6.90–14.15) degrees in glaucoma eyes, respectively. The refractive errors had a statistically significant association with horizontal LC tilt angles (coefficients, −1.53 per diopter) and glaucoma had a significant correlation with vertical tilt angles (coefficients, 6.56) using multiple logistic regression analysis (p<0.001).

**Conclusions:**

OCT allowed evaluation of the internal tilting of the LC compared with the BMO. The horizontal internal LC tilt angle was correlated with refractive errors, corresponding to myopic physiological changes, and vertical internal LC tilt was correlated with glaucoma, corresponding to glaucomatous pathological changes. These parameters have important implications for investigation of the correlation between myopia, glaucoma and LC morphological features.

## Introduction

The lamina cribrosa (LC) has been known to play a critical role in the pathogenesis of glaucoma. [1]–[3] The LC is a porous connective tissue through which retinal ganglion cell axon bundles travel in transit to the orbital portion of the optic nerve. Histopathological studies reported various changes in the LC structure, such as thinning, posterior displacement, and less connective tissue, which are thought to be associated with key mechanisms by which the retinal nerve fibers are damaged in glaucoma. [3]–[7] However, histological approaches did not allow studying the morphological dimensions of the LC in glaucomatous patients because they suffer postmortem tissue decay and dimensional changes following an intraocular pressure (IOP) decrease. Optical coherence tomography (OCT) is a noninvasive optical imaging modality that allows structural imaging of the fundus as it exists in patients. Development of spectral-domain detection technology combined with improvement of the light source to a wider wavelength range (∼50 nm) has enabled production of the currently available SD-OCT devices with 3-dimensional imaging ability and high-resolution (5–7 µm). Recent studies using SD-OCT devices revealed structural and dimensional changes associated with glaucomatous patients, such as thinning, and posterior displacement and its reversal after IOP reduction. [8]–[13] Thus, the use of SD-OCT is potentially useful for investigation of patients' LC status.

Asymmetrical deformation of the optic nerve head (ONH) in the superior-inferior and/or temporal-nasal direction may be an acquired phenomenon secondary to myopia or glaucoma. Tilted disc and peripapillary atrophy reportedly develops in association with childhood age and a greater myopic shift. [14]–[16] Park et al. reported that tilted and torsioned discs were more prevalent in myopic eyes, and the direction of the optic disc torsion was related to the location of visual field (VF) defect in patients with normal tension glaucoma based on fundus photograph analysis. [17] These interesting findings raise a question with regard to whether there is asymmetrical deformation of the LC in glaucomatous patients.

Thus, the purpose of this study was to investigate whether the vertical or horizontal LC tilt is correlated with glaucoma and/or myopia by 3-dimensional SD-OCT imaging using our novel OCT instrument with axial resolution of ∼2 µm. With the development of spectral OCT technology, improvements in axial resolution, as well as increased A-Scan acquisition rates, have enabled the visualization of 3D structures deep in the ONH, such as the LC. [18]

## Methods

The Ethics Committee of the Saitama Medical University approved this cross-sectional comparative study, which was conducted in accordance with the tenets of the Declaration of Helsinki. Patients were included if they were at least 20 years old, fulfilled the eligibility requirements detailed below and signed an informed consent at a visit from April 2012 to July 2012.

### Inclusion Criteria

To be eligible for the study, glaucoma patients had to have been previously diagnosed with primary open angle glaucoma (POAG) that fulfilled the criteria; i.e., characteristic glaucomatous ONH damage such as localized or diffuse neuroretinal rim thinning associated with glaucomatous loss of the visual field in accordance with the criteria of Anderson and Patella [19] using the Humphrey Field Analyzer (HFA; Carl Zeiss Meditec, Dublin, CA) and the standard 30-2 program of the Swedish interactive threshold algorithm (SITA).

No glaucoma subjects were enrolled during the study period. No glaucoma eyes had to have IOP between 10 mmHg and 21 mmHg, no history of glaucoma, a normal open anterior chamber angle, clinically normal appearance of the optic disc and normal visual field results.

### Exclusion Criteria

Exclusion criteria were as follows: 1) visual acuity worse than 20/40; 2) poor reliability on VF analysis (>20% fixation loss or>15% false-positive or false-negative answers); 3) any other ophthalmic disease, including media opacity, diabetic retinopathy, neuro-ophthalmological diseases, uveitis, ocular trauma, retinal or choroidal diseases, or 4) other diseases capable of causing visual field loss or optic nerve deterioration and history of intraocular surgery or laser treatment.

### Instruments

**Figure 1:**
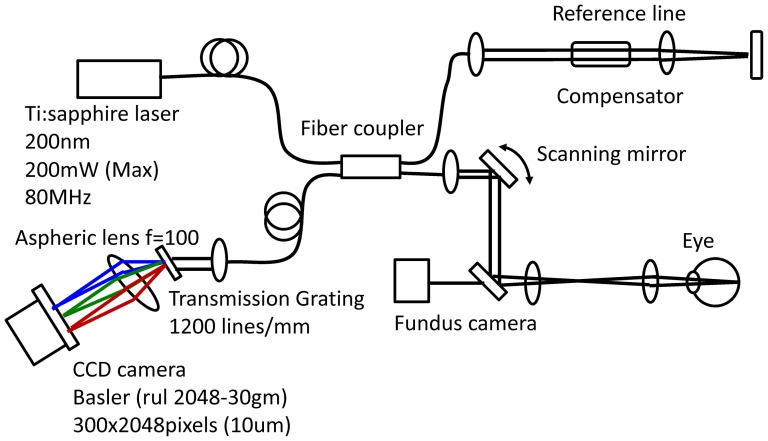
Schematic of the novel optical coherence tomography (OCT) system using a mode-locked (ML) ultra-short femtosecond laser source and a wideband spectrometer.

A schematic of the ultra-high resolution SD-OCT system is shown in Fig. 1. The OCT system was built by the Advanced Laser Medical Center (ALMC) at Saitama Medical University. The details of our SD-OCT system have been described elsewhere. [20], [21] In brief, we developed the OCT system using an ultra-broadband Kerr lens mode-locked Ti:Sapphire laser and a wideband spectrometer. The spectral bandwidth of the light source was 200 nm full-width at half maximum (FWHM) at a central wavelength of 840 nm. A high-speed CCD camera with 2048×300 pixels (Basler, Ahrensburg, Germany) was used as the detection system. The measurement speed was 50,000 depth-scans/s, and depth resolution was measured to be less than 2.0 µm into the tissue. [21] The interferometer was attached to a semi-custom fundus-scanning head system.

### Acquisition of *in vivo* three-dimensional OCT imaging

A raster scanning protocol with 300 B-scans with 300 A-scans (with 2048 pixels/A-Scan) covering a 3.0×3.0 mm square region centered at the ONH was used for volumetric scans. Volumetric rendering of the 3D-OCT data set was performed and longitudinal and *en-face* cross-sections were constructed using image processing software (Amira 5.4.3, Mercury Computer Systems Inc., Chelmsford, MA). A fundus image was generated as an *en-face* projection image from the 3D data set by integrating the magnitudes of the OCT signals at each lateral position along the axial direction. The total data acquisition time for a single 3D-OCT (volumetric) image was 3.0 seconds. OCT images were acquired after pupil dilation with tropicamide (Mydrin P; Santen, Osaka, Japan). The optic disc was also imaged by a digital 30-degree fundus camera (Zeiss FF450, Carl Zeiss, Jena, Germany) immediately before OCT data acquisition.

**Figure 2:**
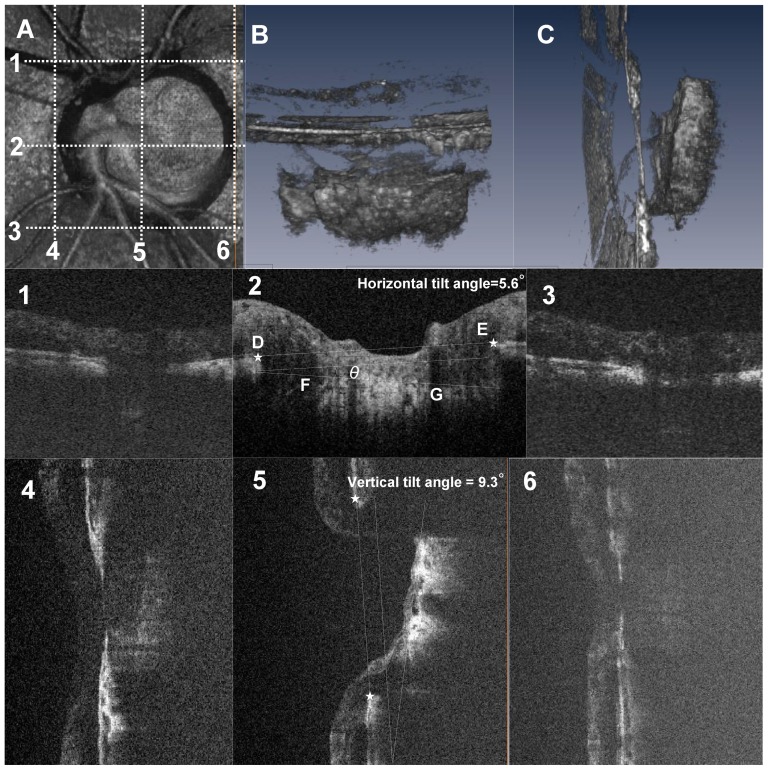
Three-dimensional spectral domain (SD) optical coherence tomography (OCT) images of the lamina cribrosa (LC) and schematic explanation of the method for measuring LC tilt angle against Bruch's membrane opening (BMO). These images are from the left eye of a 73-year-old man with normal tension glaucoma. The visual field mean deviation was −15.52 decibels. A, A *en face* view of a 3D volume rendering image including the whole optic nerve head (ONH) in a 3 mm×3 mm square area. B, C, Inferior (B) and temporal (C) view of the volume rendering ONH image. The tilting of the lamina cribrosa is evident in a vertical direction (C). Panels 1–6 are B-scan images of the scan lines indicated by dotted lines in (A). Panel 1–3, Horizontal B-scans of the ONH through the top edge (1), center (2), and bottom edge (3) of the BMO. The center scan was defined as the intermediate scan between the superior (1) and inferior (2) scans. The lamina tilt angle (angle θ) was defined as the angle between the reference plane (BMO plane, line DE) and the anterior LC plane (line FG) at the center B-scan image. The horizontal tilt angle was 5.6 degrees in this case. 4–6, Vertical B-scan of the ONH at the nasal edge (4), center (5), and temporal edge (6) of BMO. The vertical tilt angle was 9.3 degrees in this case.

Fig. 2 shows a diagram of the creation of the image of the optic disc obtained with the OCT instrument. Fig. 2B and 2C are 3D volume rendering images of the ONH.

### LC Tilt Angle Measurement

**Figure 3:**
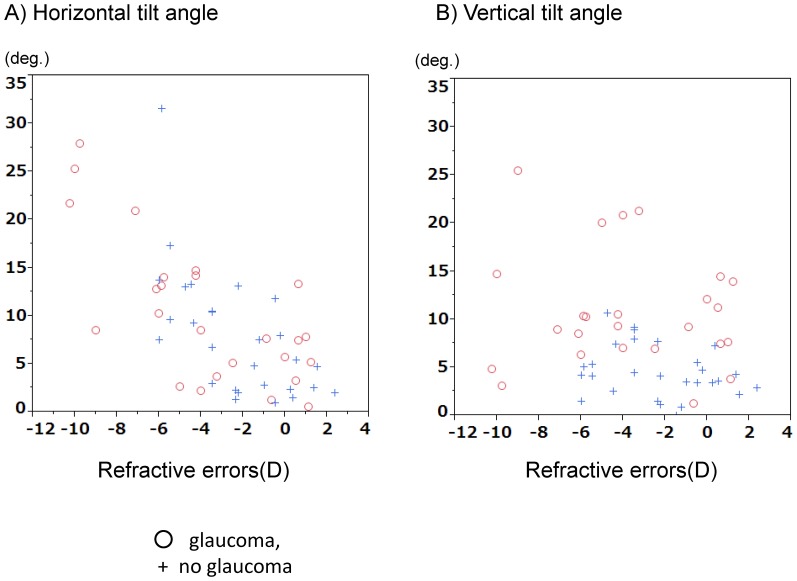
The scatterplot graphs of the lamina cribrosa tilt angles and refractive errors (spherical equivalent). Red circle, with glaucoma; blue cross, without glaucoma. (Left) Correlation between horizontal lamina tilt angles and refractive errors. (Right) Correlation between vertical lamina tilt angles and refractive errors.

**Figure 4:**
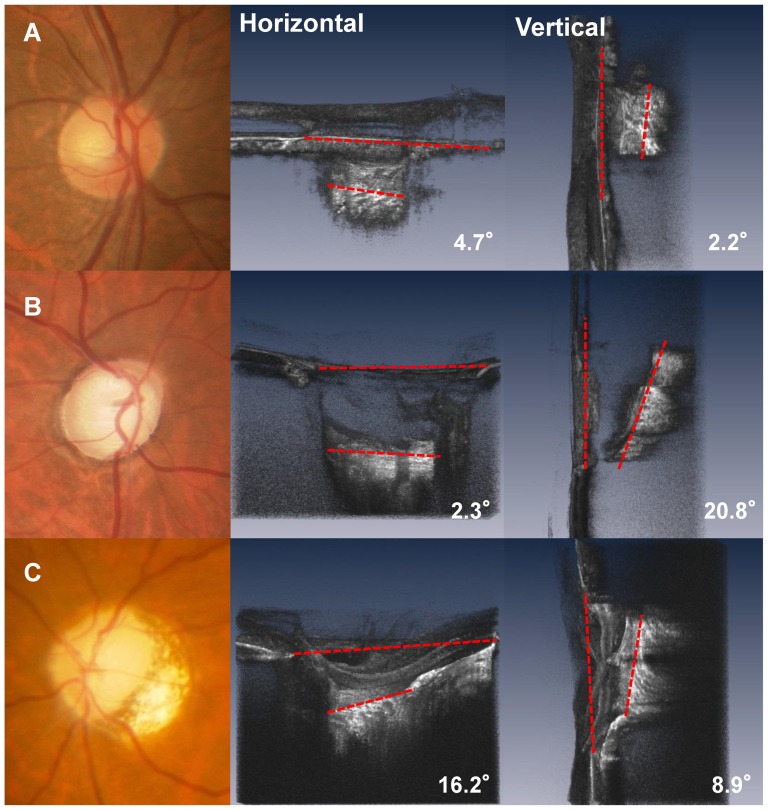
Fundus image and horizontal and vertical optical coherence tomography (OCT) images of the lamina cribrosa (LC). A. (Top left) images are from the right eye of a 63-year-old man without glaucoma. The refractive error was −0.5D. (Top center and Top right) The LC tilting is mild in both horizontal and vertical directions. B. (Middle left) Images are from the right eye of a 68-year-old man with glaucoma. The refractive errors were −1.25D and the visual field mean deviation was −18.22 decibels. Inferior (middle center) and nasal (middle right) view of the volume rendering ONH image. In contrast to his horizontal LC tilt angles (2.3°), the vertical LC tilt angle was much greater (20.8°). C. (Bottom left) Images are from the left eye of a 54-year-old woman with glaucoma with a high myopic eye. Inferior (Bottom center) and temporal (Bottom right) view of the volume rendering ONH image. The refractive errors were −7.2D and the visual field mean deviation was −11.33 decibels. Both horizontal (16.2°) and vertical LC (8.9°) tiltings are evident.

After acquisition an *in vivo* 3D dataset, B-scan images were horizontally and vertically reviewed. Fig. 2 shows a schematic explanation of the method for measuring the LC tilt angle against the Bruch's membrane opening (BMO) and we defined horizontal and vertical LC tilt angles as follows. The horizontal center of the B-scan (Fig. 2-2) was defined as the intermediate scan between the superior (Fig. 2-1) and inferior (Fig. 2-3) scans in which interruption of the Bruch's membrane line started. The vertical center of the B-scan (Fig. 2-5) was defined as the intermediate scan between nasal (Fig. 2-4) and temporal (Fig. 2-6) scans in which disruption of the Bruch's membrane line started.

A line (line DE) connecting the proximal tips of Bruch's membrane on each side of the ON head on the cross sectional SD-OCT image (star points D and E, Fig. 2-2) was drawn and set as the BMO reference plane. The LC tilt angle (angle θ) was defined as the angle between the reference plane (line DE) and approximate the best-fitting line for the anterior LC plane (line FG). The anterior surface of the LC on B-scans was defined as the location where high reflectivity started beneath the optic disc cup. Angle measurements were performed with Amira software. If eyes show W-shape anterior LC surface, the observer drew straight-line approximation.

The measurements were repeated three times and averaged to provide the final LC tilt angle. The intraobserver and interobserver reproducibility of LC tilt measurements were evaluated in 30 randomly selected eyes from 15 glaucoma eyes and 15 normal eyes. Analysis was based on three independent series of re-evaluations made by two independent observers. The absolute agreement of a single observer's measurement and the mean of all three measurements conducted by the two observers were calculated with the intraclass correlation coefficient (ICC) using a 2-way mixed effect model.

### Statistical Analyses

Data are presented as mean and standard deviation (SD), median with interquartile range for continuous variables or as a frequency with percentage for categorical variables. Baseline characteristics were summarized by treatment sequences and compared using an unpaired *t* test, the Wilcoxon rank sum test for continuous variables and a chi square test or Fisher's exact test for categorical variables as appropriate. Because some subjects had both eyes enrolled in the study, the generalized estimating equation (GEE) was used when analyzing data derived from eyes (as opposed to people).

To determine the effects of various factors on the horizontal and vertical LC tilt angle, we performed univariate and multivariate regression analyses. The model was adjusted with candidate factors including age, sex, glaucoma and refractive errors. Coefficients with 95% confidence intervals (95% CI) are presented. A *P*-value less than 0.05 indicated a statistically significant difference. All statistical analyses were performed using JMP version 10.1 software (SAS Institute, Inc., Cary, NC, U.S.A.) SAS ver. 9.3 software (SAS Institute Inc., Cary, NC, U.S.A).

## Results

During the enrollment period, this study initially involved 57 eyes of 34 patients. Of these eyes, we excluded 4 eyes with improper OCT images due to media opacities and inappropriate fixation. Thus, 53 eyes (from 31 patients) were included in this study; 28 no glaucoma eyes and 25 eyes with glaucoma. Table 1 summarizes the baseline characteristics of the study subjects. The mean age ± standard deviation was 51.0±17.3 years in eyes without glaucoma, and 63.2±8.7 years in eyes with glaucoma; this difference was statistically significant. The mean spherical equivalent error ± standard deviation was −2.3±2.5 (range −6.00 to +2.38) diopters in eyes without glaucoma, and −3.7±3.8 (range −10.63 to +1.25) diopters in eyes with glaucoma; this difference was not statistically significant. Our study included eyes with varying extent of tilted disc, but not with tilted disc syndrome (inferiorly tilted disc).

**Table 1:** Baseline characteristics of the group without glaucoma and and the group with glaucoma.

	*no glaucoma group*			*glaucoma group*			*P value*
	*(n = 28)*			*(n = 25)*			
*Male (n, %)*	21		(75.0)	13		(52.0)	0.080
*Age (yrs)*	51.0	±	17.0	63.2	±	8.7	0.002
*Spherical equivalent error (D)*	−2.3	±	2.5	−3.7	±	3.8	0.103
*(range)*	(−6.00 to +2.38)			(−10.63 to +1.25)			

Plus-minus values are means ± SD.

Baseline characteristics were compared with the unpaired t-test between the groups.Abbreviations: yrs, years; MD, mean deviation; dB, decibels;

D, dioptres.

Mean LC tilt angle measurements showed excellent intraobserver (ICC  = 0.973 for observer 1 and ICC  = 0.972 for observer 2) and interobserver (ICC = 0.968) agreement (all P<0.001). The median (interquartile range, [IQR]) horizontal and vertical LC tilt angles were 7.10 (2.43–11.45) degrees and 4.15 (2.60–6.85) degrees in no glaucomay eyes, and 8.50 (4.40–14.10) degrees and 9.30 (6.90–14.15) degrees in eyes with glaucoma, respectively (Table 2). Fig. 3A and 3B show the scatterplot graphs of the refractive errors and horizontal and vertical tilt angles. Greater horizontal tilt angles were significantly correlated with severer refractive errors both in the control group (r = −0.639, p<0.001) and the glaucoma group (r = −0.725, p<0.001), but no significant correlations were found between vertical LC tilt angles and refractive errors. Fig. 4 shows a representative photograph. We confirmed that horizontal LC tilt was greater in high myopic eyes (patient C) than emmetropic no glaucoma (subject A) and glaucoma (patient B) eyes. In contrast, vertical tilt angles were greater in glaucoma eyes (patient B and C) than no glaucoma eyes (subject A). Table 3 describes the results of univariate and multivariate analyses for factors potentially affecting LC tilt angles. In univariate analysis, greater refractive errors had a statistically significant association with horizontal LC tilt angles, and the presence of glaucoma was associated with greater vertical LC tilt angles. After adjusting for other potential factors using multivariate analysis, the greater refractive errors were significantly correlated with horizontal LC tilt angles (coefficient, −1.53 per diopter; *p*<0.001) and the presence of glaucoma was correlated with greater vertical LC tilt angles (coefficient, 6.56; p<0.001). The interaction between refractive errors and glaucoma did not show statistically significant correlations with vertical tilt angles in multivariate analyses.

**Table 2:** Horizontal and vertical lamina cribrosa tilt angles in each group.

	*no glaucoma group*	*glaucoma group*	*P value*
	*(n = 28)*	*(n = 25)*	
*Horizontal tilt angle (deg)*	7.10 (2.43, 11.45)	8.50 (4.4, 14.10)	0.179
*Vertical tilt angre (deg)*	4.15 (2.60, 6.85)	9.30 (6.95, 14.15)	<0.001

median(25, 75th percentile).

Abbreviations: deg., degrees.

**Table 3:** Association of Potential Clinical and Biometric Parameters With the Lamina Tilt Angle Based on Univariate and Multivariate Analyses in 53 Eyes From 31 Control Subjects and Glaucoma Patints.

	*horizontal tilt angle*				*vertical tilt angle*			
*Variables*	*Univariate*		*Multivariate*		*Univariate*		*Multivariate*	
	*Coefficients (95%CI)*	*P Value*	*Coefficients (95%CI)*	*P Value*	*Coefficients (95%CI)*	*P Value*	*Coefficients (95%CI)*	*P Value*
***Age (per y)***	−0.08 (−0.19, 0.03)	0.157	0.00 (−0.07, 0.07)	0.991	0.06 (−0.04, 0.17)	0.207	−0.02 (−0.11, 0.06)	0.575
***Sex (reference: male)***	0.88 (−2.72, 4.49)	0.631	−1.00 (−3.88, 1.87	0.494	0.48 (−2.77, 3.72)	0.774	−1.56 (−4.35, 1.24)	0.275
***Glaucoma (reference: normal)***	2.47 (−1.17, 6.11)	0.184	0.49 (−2.37, 3.34)	0.739	6.20 (3.73, 8.67)	<0.001	6.56 (3.19, 9.93)	<0.001
***Refractive errors (SEQ) (per diopter)***	−1.52 (−1.88,−1.17)	<0.001	−1.53 (−1.96, −1.11)	<0.001	−0.43 (−0.81, −0.05)	0.027	−0.20 (−0.56, 0.15)	0.254

Abbreviations: SEQ, spherical equivalent

## Discussion

To the best of our knowledge, this is the first study that showed LC tilt angles against BMOs can be measured by 3D imaging using SD-OCT and this new LC parameter correlated with clinical factors. The measurement was possible because SD-OCT can depict the surface of the LC and BMO. We used the BMO to define the LC tilt angle, but not the photographic disc margin, because photographically determined clinical disc margins reportedly represent complex anatomical structures that differ in individual eyes. [22]–[24] The 3D imaging allowed measurement of the LC angle along the horizontal and vertical lines through the optic disc center, which was able to be determined by using the temporal, nasal, superior and inferior edges of the BMO on the 3D dataset as references.

The principle findings of this report were that both refractive errors and the presence of glaucoma were significantly related to greater LC tilt angles, and the effects of these 2 factors on LC tilting differed with the tilting direction; larger refractive errors were correlated with greater horizontal LC tilt angles and the presence of glaucoma was significantly correlated with larger vertical LC tilt angles. Thus, the clinical relevancy of LC tilting may be different with different tilting directions.

Using EDI-OCT, Park et al. [25] reported that the anterior LC surface was more posteriorly located in the superior and inferior regions than in the nasal and temporal regions in healthy human subjects. Thus, the LC structure has normal regional differences, which may underline the different results between vertical and horizontal LC tilting in our study. *In vivo* human imaging studies showed various abnormal changes of the LC structure in glaucomatous eyes, such as thinning [11]–[13], and posterior displacement [8] of the LC. These findings are consistent with those of previous studies using histological specimens [3], [6], [7], [26] and computerized modeling. [27], [28] Although the LC tilting we measured in this study using OCT has not been histopathologically reported in human eyes, the greater LC tilting in glaucomatous eyes found in our study may be related to the posterior displacement of the LC. Recently, Yang et al. [29], using early experimental glaucoma monkey eyes, showed that posterior migration of both the anterior and posterior LC insertion was a component of early glaucomatous cupping. Thus, it appears that posterior migration of LC insertion as well as well-known LC changes, such as thinning, and backward bowing [30] taken together leads to posterior displacement. Our findings suggest that posterior displacement occurs asymmetrically between superior and inferior regions; but it remains unknown which components of LC changes are responsible for the greater vertical LC tilting in glaucoma.

It was also shown in histopathological studies using human specimens that the superior and inferior parts of the LC suffer from greater compression and backward bowing than the middle region of the LC in glaucoma. [6], [7] Such regional differences in LC changes has been attributed to histopathological findings such that the superior and inferior parts of the LC have a lower density of connective tissue and larger laminar pores than the nasal and temporal parts. These differences in superior and inferior LC structure are thought to be associated with greater vulnerability to glaucomatous damage of the retinal nerve fibers passing through the superior and inferior LC. [6] Although it still remains unknown whether the superior and inferior parts of the LC suffer from greater posterior migration of the LC insertion, the susceptibility of the superior and inferior LC to structural changes associated with glaucoma may be responsible for our finding that vertical LC tilting, but not horizontal LC tilting, was associated with the presence of glaucoma.

Myopia has been reported to be related to optic disc tilting. [31] Recently, Kim et al. showed that the tilted disc in myopic eyes may be an acquired feature caused by scleral stretching in the parapapillary region associated with a myopic shift. [14] In this study, horizontal, but not vertical, LC tilting was correlated with refractive errors. Interestingly, our results are consistent with previous findings in that structural changes in the horizontal direction are associated with myopia regardless of the fact that LC tilting and optic disc tilting are not identical. We used the BMO as a reference plane to measure LC tilting. However, the BMO, as well as scleral openings, is also tilted probably in association with optic disc tilting in myopic eyes. It needs to be determined whether optic disc tilting and LC tilting in the horizontal direction in myopia are dependent or independent phenomena. Our findings, taken together with the previous findings, at least suggest that the effects of myopia on the optic disc structure are not only on the BMO but also on the LC. Several limitations warrant discussion. First, this was a pilot study with a small number of subjects. Second, the control group was not age-matched with the glaucoma group. To control the effects of age differences, we adjusted for potential factors, including age, using multivariate analysis. Further study using a greater number of age-matched cases will be necessary to confirm our findings. Third, we measured LC tilting within the optic disc cupping because the reflectivity of the LC beneath the neuroretinal rim was weaker than that within the cupping. To exclude the possibility of incorrect measurement, we did not use the LS surface beneath the rim. Fourth, we could not correct magnification effects in OCT imaging on lateral measurement of fundus structures, which affect the angle measurement results. Therefore, the possibility cannot be excluded that the magnification effects were, at least in part, responsible for the correlation between horizontal tilting and refractive errors. However, this would not be critical in our results because refractive errors were associated only with horizontal tilt angles and with vertical tilt angles in multivariate analysis regardless of the identical magnification effects on horizontal and vertical measurements.In conclusion, we obtained *in vivo* 3D images of the LC and measured its horizontal and vertical tilt angles in patients with and without glaucoma. The horizontal LC tilt angle was correlated with refractive errors, and vertical LC tilt angle was correlated with glaucoma. These results suggest that the tilting of LC against the BMO are multifactorial; i.e., affected by at least two factors. Further studies are required to clarify the internal structural changes of the optic disc associated with glaucoma.
